# Study of Streptococcus mutans in Early Biofilms at the Surfaces of Various Dental Composite Resins

**DOI:** 10.7759/cureus.38090

**Published:** 2023-04-25

**Authors:** Dhaifallah Alqarni, Masatoshi Nakajima, Junji Tagami, Mohammed S Alzahrani, Ana Clara Sá-Pinto, Ali Alghamdi, Keiichi Hosaka, Fouad Alzahrani, Omar A Alsadon, Raed A Alharbi, Shaia S Almalki, Abdullah Ali H Alzahrani

**Affiliations:** 1 Restorative and Prosthodontic Department, Almikhawah Dental Center, Al-Baha, SAU; 2 Department of Cariology and Operative Dentistry/Oral Health Sciences, Graduate School of Medical and Dental Sciences, Tokyo Medical and Dental University, Tokyo, JPN; 3 Restorative Dental Sciences Department, School of Dentistry, Al-Baha University, Al-Baha, SAU; 4 Department of Pediatric Dentistry and Orthodontics, School of Dentistry, Universidade Federal dos Vales do Jequitinhonha e Mucuri, Minas Gerais, BRA; 5 Department of Regenerative Dental Medicine, Graduate School of Biomedical Sciences, Tokushima University, Tokushima, JPN; 6 Pulp Biology and Endodontic Department, Al-Baha Dental Center, Al-Baha, SAU; 7 Department of Dental Health, School of Applied Medical Sciences, King Saud University, Riyadh, SAU; 8 Department of Laboratory Medicine, School of Applied Medical Sciences, Al-Baha University, Al-Baha, SAU; 9 Department of Dental Health, School of Applied Medical Sciences, Al-Baha University, Al-Baha, SAU

**Keywords:** dentistry, dental composite resins, surfaces, biofilms, streptococcus mutans

## Abstract

Background: Biofilm deposit on the composite restoration is a common phenomenon and bacterial growth follows the deposition. The study aims to evaluate *Streptococcus mutans* (*S. mutans*) early biofilm formation on the surfaces of various dental composite resins by using the real-time quantitative polymerase chain reaction (qPCR) technique.

Materials and methods: Thirty-two discs, where eight discs were in each group of Filtek Supreme Ultra (FSU; 3M, St. Paul, MN), Clearfil AP-X (APX; Kuraray Noritake Dental Inc., Tokyo, Japan), Beautifil II (BE2; Shofu, Inc., Kyoto, Japan), and Estelite Sigma Quick (ESQ; Tokuyama Dental, Tokyo, Japan), were fabricated and subjected to *S. mutans* biofilm formation in an oral biofilm reactor for 12 hours. Contact angles (CA) were measured on the freshly fabricated specimen. The attached biofilms underwent fluorescent microscopy (FM). *S. mutans* from biofilms were analyzed using a qPCR technique. Surface roughness (Sa) measurements were taken before and after biofilm formation. Scanning electron microscopy (SEM), including energy dispersive X-ray spectrometer (EDS) analysis, was also performed for detecting relative elements on biofilms.

Results: The study showed that FSU demonstrated the lowest CA while APX presented the highest values. FM revealed that condensed biofilm clusters were most on FSU. The qPCR results indicated the highest *S. mutans* DNA copies in the biofilm were on FSU while BE2 was the lowest (p < 0.05). Sa test signified that APX was significantly the lowest among all materials while FSU was the highest (p < 0.05). SEM displayed areas with apparently glucan-free *S. mutans* more on BE2 compared to APX and ESQ, while FSU had the least. Small white particles detected predominantly on the biofilms of BE2 appeared to be Si, Al, and F extruded from the resin.

Conclusion: Differences in early biofilm formation onto various composite resins are dependent on the differences in material compositions and their surface properties. BE2 showed the lowest quantity of biofilm accumulation compared to other resin composites (APX, ESQ, and FSU). This could be attributed to BE2 proprieties as a giomer and fluoride content.

## Introduction

When it comes to endodontics, the wettability of the adhesive and the contact angle it has with the enamel surface are both heavily influenced by the quantity and quality of the etching pattern that is produced when acids are used. When the etch pattern is better, the enamel will have a higher surface energy, which will improve the adhesive's ability to penetrate the enamel and will, as a result, result in a stronger bond.

Reclamation of masticatory and esthetic functions calls for the utilization of appropriate and quality dental restorative materials [1]. Nonetheless, these therapeutic products are susceptible to the formation of biofilms leading to adverse oral health outcomes [2,3]. In dentistry, restorative materials such as composite resins have been commonly used for their esthetic, longevity, and minimally invasive preparations. However, there are still quite a few disadvantages to their use, such as polymerization shrinkage and susceptibility to recurrent caries [4]. Furthermore, with the consequential growth in plaque accumulating on restoration surfaces, the surrounding tooth structures will be at high risk of demineralization [5]. When compared to other restorative materials, composite restorations are more vulnerable to bacterial attachment than other materials, such as dental ceramics and metallic alloys [6]. Bacterial colonization or biofilm formation leads to secondary caries at the restoration margins, affecting the longevity of the restoration [7]. The type and quantity of organic matrix and inorganic filler particles present in the restorative materials determine the physical and chemical characteristics, which consequently influence the structural and handling properties of resin composite [8]. Alongside the hardness, resistance to wear, and mechanical and esthetic properties resulting from this composition, these components as well play a major role in the bonding to other materials in case of repair and influence the bacterial biofilm accumulation, particularly resin monomers release [9,10].

Several studies [1,11-14] have linked the surface roughness of restorative materials to bacterial adhesion. Smooth resin composite surfaces were shown to have less bacterial adhesion and accumulation than rougher resin composite surfaces [1]. A different investigation showed that smooth resin composite surfaces have a significant effect in preventing biofilm adhesion and development [13]. Proximal restorations, however, are still out of reach of adequate mechanical polishing, thus they must rely on plastic matrix strips to automatically produce smooth surface layers made mostly of resin. Chemical makeup, surface roughness, and wettability [12], all have a role in establishing the topography of composite resins. First, bacteria adhere to the solid surface of the substrate [15,16], which may be tooth structure or composite restoration. The first germs to colonize a tooth tend to settle along the enamel's fissures and ridges [12,17], as shown by studies of early plaque adherence to tooth surfaces. In the same way, the rough surfaces of dental composites play a crucial role in the onset of bacterial adherence to the materials in the mouth cavity.

It is well established that the adherence of mutans streptococci to the surface of a tooth or other solid structures in the oral cavity is the first step in the sequential process of cariogenic biofilm development, just as it is with the production of other types of dental plaque. The early colonization of the host tissue surfaces and all exposed dental material surfaces by oral bacteria is considered a crucial phase. Cariogenic bacteria are common, but *Streptococcus mutans* (*S. mutans*) stand out as the most dangerous among the mutans streptococci. The synthesis of water-insoluble glucan (WIG) from sucrose is mediated by the glucosyltransferases (gtf) genes of *S. mutans* [18]. Principally, WIG serves as the bacterial extracellular polymeric substance (EPS) in oral biofilms and plays a major role in biofilm condensation, cell-to-cell cementation, growth, and maturation [19].

The oral biofilm reactor (OBR) has been effectively employed for the formation of cariogenic biofilms in vitro mostly on restorative materials. However, there are few investigations that have evaluated the early stage of *S. mutans* biofilm formation onto the cut filler surface of composite resin restorations. From a dental public health perspective, exploring and investigating the early biofilm formation of *S. mutans* on different cured dental composite surfaces may be meaningful. This is mainly because *S. mutans* has been considered one of the most important causes of the prevalence of dental caries worldwide. *S. mutans* has not only been shown to adhere the microorganisms to the tooth enamel, but also it has produced a significant element in the colonization of *S. mutans* in dental biofilm, which is mutacins (bacteriocins) [20,21]. This study aimed to evaluate *S. mutans* early biofilm formation on the cured dental composite resin surfaces of four different composite resin restorative materials by using the real-time quantitative polymerase chain reaction (qPCR) technique. The null hypothesis was that the biofilm formation will be similar on the surfaces of four different composite resins.

## Materials and methods

Specimen preparation

Only one person was involved in the process to reduce the bias. Four dental resin composites were used in the present study (Table 1).

**Table 1:** Compositions of the dental composite resin materials used in this study bis-GMA: bisphenol A-glycidyl methacrylate; TEGDMA: triethylene glycol dimethacrylate; UDMA: urethane dimethacrylate; bis-EMA: bisphenol A-ethoxylated dimethacrylate; PEGDMA: polyethylene glycol dimethacrylate.

Materials	Manufacturer	Compositions
Clearfil AP-X (APX)	Shade A2, Kuraray Noritake Dental Inc., Tokyo, Japan	Barium glass filler, colloidal silica fillers, filler load 85 wt% (71 vol%), bis-GMA, TEGDMA
Beautifil II (BE2)	Shade A2, Shofu, Inc., Kyoto, Japan	Aluminofluoro-borosilicate glass, S-PRG fillers, filler load 83.3 wt% (68.6 vol%), bis-GMA, TEGDMA
Estelite Sigma Quick (ESQ)	Shade A2, Tokuyama Dental, Tokyo, Japan	Silica–zirconia fillers, silica-titania fillers, fillers load 82 wt% (71 vol%), bis-GMA, TEGDMA
Filtek^TM^ Supreme Ultra Universal restorative (FSU)	Shade A2, 3M, St. Paul, MN, USA	Non-agglomerated/non-aggregated zirconia, silica, aggregated zirconia/silica clusters, filler load 78.5 wt% (63.3 vol%), bis-GMA, UDMA, TEGDMA, bis-EMA, PEGDMA

**Figure 1:**
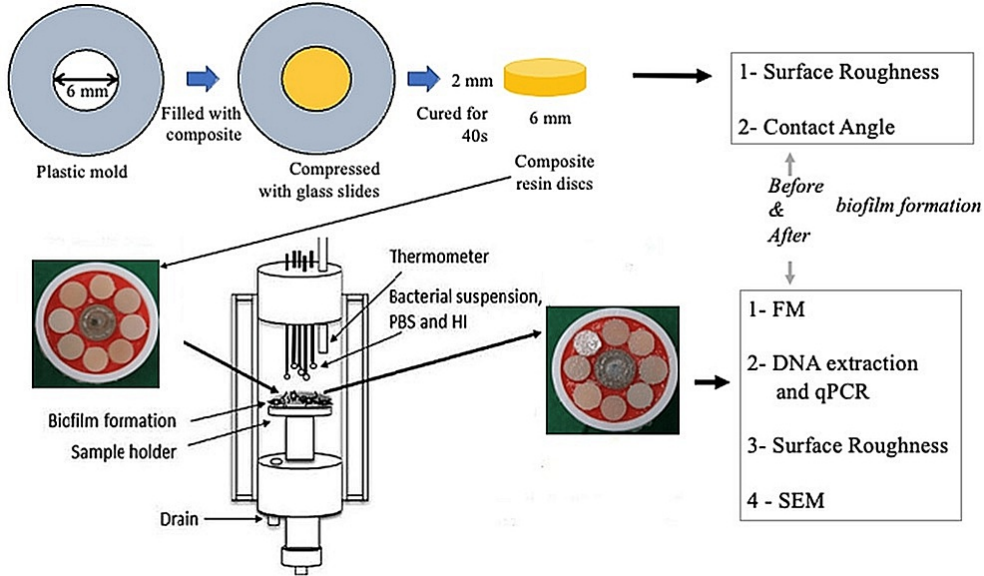
Summary of all experimental and analysis procedures conducted in this study PBS: phosphate-buffered saline; HI: heart infusion broth; FM: fluorescent microscopy; qPCR: quantitative polymerase chain reaction; SEM: scanning electron microscopy.

The key reason for selecting these four dental composites is centered on the fact that they have different characteristics, which may influence biofilm formation. For example, Clearfil AP-X (APX; Kuraray Noritake Dental Inc., Tokyo, Japan) has irregular fillers, Filtek Supreme Ultra (FSU; 3M, St. Paul, MN) has nano-fillers and clusters, Estelite Sigma Quick (ESQ; Tokuyama Dental, Tokyo, Japan) has a smooth surface, and finally, Beautifil II (BE2; Shofu, Inc., Kyoto, Japan) contains fluoride and is considered a glass ionomer resin. A total of 32 discs were prepared. The power value was calculated to be equal to 95, and the sample size was determined to be 32, with four groups of etchants and eight samples in each group. As shown in the upper part of Figure 1, which summarizes all trial and examination methods used in this review, a straightforward plastic grid strip (Hawe Striproll Straightforward, 10 mm, KerrHawe, Bioggio, Switzerland) was placed on the glass slide, and a round shape (6 mm in width and 2 mm in thickness) was set on it.

When the molds were filled with gum composite, a second plastic grid strip was placed on top of each sample, and a thin covering glass slip (0.1 mm thick) was applied to the top of each to apply a gentle strain and create a flat surface. Each plate was exposed to a light source with an intensity of more than 600 mW cm^2^ for 40 seconds using an incandescent lamp fix unit (Optilux 501, Kerr, Orange, CA). Smart removal of glass slip and plastic strip, as well as the elimination of all light-restored gum composites, allowed us to reuse the molds.

Biofilm formation

For this research, *S. mutans* MT8148 was employed. *S. mutans* was washed many times and then suspended in phosphate-buffered saline (PBS) using bacteria that had been newly grown for 16 hours in brain/heart infusion broth (Becton Dickinson, Sparks, MD). The *S. mutans* suspension was maintained at 4°C. Using heart infusion (HI) broth (Becton Dickinson, Sparks, MD), a solution of sucrose (0.1% final concentrations) was produced and autoclaved for use in in-vitro biofilm growth. The biofilm was grown using an oral biofilm reactor (OBR), with the help of two identical jacketed chambers where the composite discs were put on top of red utility wax (GC, Tokyo, Japan). There were five 21-gauge stainless steel tubes in each chamber that were sealed with a silicon stopper, turning each into an incubator that maintained a constant 37°C. Steel tubing had its other end joined to silicon tubing from pumps activated by a computer-controlled controller (EYELA EPC-2000, Tokyo Rika, Tokyo, Japan). If you run liquids through tubes long enough, they will create domes that evenly cover the resin composite samples. As the domes reached their maximum height, the liquid overflowed and was drained away, with the pH reading from the bulb electrode being continually recorded. Each specimen with biofilm built on was removed after a cycle of 12 hours inside the OBR. It could be highlighted that sterilizing the OBR machine was conducted by running the system with 99% alcohol for at least one hour to ensure that every tube and surface in contact with later fluids in the system tract was sterilized.

Contact angle (CA) measurement

Discs from each composite were divided into two groups depending on the liquid type: (A) deionized water and (B) acidic liquid with pH5. Each liquid was represented by a droplet that was deposited on the surface of the specimen and the contact angle was measured (VCA Optima XE, Techscience, Koshigaya, Japan). Constantly observing the drop's surface allowed the researcher to determine the contact angle at 20, 40, and 60 seconds after the droplet had settled. Each measurement was repeated four times by the same researcher, and the mean value was reported.

Surface roughness (Sa) analysis

Each surface was measured in four distinct spots chosen at random using a laser scanning confocal microscope (VK-X150, KEYENCE Co., Ltd, Osaka, Japan). We were able to get some standard pictures of three-dimensional (3D) surface topography and measure the 3D surface roughness parameter Sa (arithmetic mean deviation).

Fluorescent microscopy (FM)

After biofilms formed on the resin disc surfaces, two discs from each composite with the attached biofilms were stained immediately with LIVE/DEAD^TM^ BacLight Bacterial Viability Kit (Thermo Fisher Scientific, Waltham, MA), a mixture of SYTO 9, and propidium iodide (PI) (Molecular Probes, Invitrogen Detection Technologies, Carlsbad, CA) after lightly washing in PBS and subjected to fluorescence microscopy (FM; CKX41, Olympus, Tokyo, Japan). The attached biofilms were photographed using x10 and x100 objective lenses immediately after exciting with blue and green fluorescent lights subsequently on the same microscopic field of view after randomly selecting four different fields.

Extraction of DNA from the biofilms

All biofilms were gently wiped and removed from each sample by using sterilized small-size sponges. Using a DNeasy tissue device (QIAGEN, Tokyo, Japan), genomic DNA was extracted and destroyed from disposable wipes. Each sample was centrifuged to kill bacteria (at 20,000 g for 10 minutes at 4°C), resuspended in 570 l of a 20 mg ml1 lysosome (Sigma, Tokyo, Japan) solution containing 50 mM Tris-hydrochloride (pH 8.0) and 20 mM ethylenediaminetetraacetic acid (EDTA), and incubated at 37°C for 30 minutes to hatch. Next, we added 30 l of a solution containing 20 mg/mL Proteinase K (QIAGEN) and incubated the whole thing at 55°C for 10 minutes to hatch the example. Tissue Lyser (QIAGEN) shakes the samples at 30 Hz for 10 minutes after adding 0.8 g of corrosive-washed glass dots (Sigma) and 1.5 l of 100 mg ml1 RNase A. After that, we added 600 l of cradle AL (lysis cushion) from a DNeasy tissue pack (QIAGEN) and brooded it at 70°C for 30 minutes. The supernatants were transferred to fresh cylinders and the glass dots were lowered. After adding 500 liters of 99.5% ethanol (WAKO, Tokyo, Japan) and spinning at 5800 rpm and 4°C for a minute, we had a look at the liquid. After adding 500 liters of AW1 (wash buffer 1, QIAGEN), the mixture was centrifuged (5800 g at 4°C for two minutes). After that, we added 500 liters of AW2 (wash buffer 2, QIAGEN) and spun the mixture at 20,000 revolutions per minute at 4°C for two minutes. The last steps included adding 200 l of cradle AE (elution support, QIAGEN), waiting a little while at room temperature, and then centrifuging (5800 g at 4°C for one second).

Quantitative real-time PCR (qPCR)

A real-time qPCR experiment was performed on genomic DNA from each sample for quantitative detection of *S. mutans* using the ABI PRISM 7500 equipment (Applied Biosystems, Waltham, MA). The sequences of primers used are Sm-F5; 5’- AGCCATGCGCAATCAACAGGT-3’and Sm-R4; 5’-CGCAACGCGAACATCTTGATCAG-3’ [22]. Twenty-five microliters of SYBR Green PCR master mix (Applied Biosystems), 20 μl of the extracted DNA template from each sample, 2 μl of each primer, and 1 μl of nuclease-free water were used in each reaction tube. Ten minutes of activation at 95°C was followed by 40 cycles of 15 seconds of denaturation at 95°C, one minute of primer annealing, and extension at 68°C, all performed in a thermal cycler. DNA from the same strain of *S. mutans* was maintained in ready stock at -25°C and a standard curve was constructed on the ABI PRISM 7500 system at regular intervals using a 10-fold dilution series. The absence of nucleases in water was employed as a negative control. The data were analyzed using Sequence Detection Software, version 1.6.3. (Applied Biosystems). To account for the entire elute volume from each specimen surface area, the raw qPCR results were multiplied by 10 (28.3 mm^2^). The number of cells in the biofilm generated on the resin-composite surfaces after 12 hours was given as the number of DNA copies.

Scanning electron microscopy (SEM) and energy dispersive X-ray spectrometer (EDS) observation and analysis

Two samples were chosen at random from each demineralized epoch and prepared for SEM analysis. After overnight incubation at 4°C in 1% glutaraldehyde, specimens were rinsed in PBS and placed in a desiccator to fully remove all traces of moisture. A scanning electron microscope (SEM, JSM-5310LV, JEOL, Tokyo, Japan) was used to look at the biofilms that had been adhered after they had been coated in gold (SC-701AT, Elionix, Tokyo, Japan). Both x1000 and x2000 magnification photographs were then taken. Chemical components, including Si, Al, and F, that seemed to have extruded mostly from BE2 through to the biofilm surfaces were also detected using a scanning electron microscope (SEM)-energy dispersive X-ray spectrometer (EDS) (Hitachi S-4500, Hitachi, Tokyo, Japan).

Statistical analysis

SPSS version 15.0 was used for all numerical data analysis (SPSS Inc., Chicago, IL). Two-way analysis of variance (ANOVA) was used to compare contact angle and surface roughness data across groups, while one-way ANOVA was used to compare qPCR data between groups at a 95% confidence level, with Bonferroni correction used.

## Results

Contact angle (CA)

The results of the contact angle data are displayed in Table 2.

**Table 2:** Mean and standard deviation values of contact angle measurements

Water/PH per second	Beautifil II (BE2)	Clearfil AP-X (APX)	Filtek Supreme Ultra (FSU)	Estelite Sigma Quick (ESQ)	p-value
Water/20 s	62.7 ± 0.1	68.6 ± 1.3	57.7 ± 0.3	62.5 ± 0.2	<0.001
Water/40 s	60.7 ± 0.1	66.4 ± 1.9	56.1 ± 0.7	59.9 ± 0.9	<0.001
Water/60 s	59.1 ± 0.5	64.2 ± 3.1	54.0 ± 1.0	58.1 ± 0.5	<0.001
PH5/20 s	60.8 ± 0.4	65.6 ± 3.2	62.6 ± 0.5	62.7 ± 0.1	<0.001
PH5/40 s	59.6 ± 0.8	64.1 ± 3.5	57.9 ± 0.2	61.6 ± 0.1	<0.001
PH5/60 s	57.3 ± 0.9	63.3 ± 2.3	56.9 ± 0.4	60.6 ± 0.1	<0.001

FSU showed the lowest contact angle in both water as well as in an acidic medium after 20 seconds, 40 seconds, and 60 seconds, whereas the highest contact angle in both water and an acidic medium after 20 seconds, 40 seconds, and 60 seconds was seen in APX. The overall difference in contact angles in both media among the four groups was significant.

APX showed a significantly higher contact angle as compared to other materials in both water and PH5 media at each time interval tested. FSU showed a significantly lower contact angle than BE2 material in water but an almost similar contact angle in the acidic medium at each time interval. BE2 showed a significantly lower contact angle as compared to ESQ only in an acidic medium only after 60 seconds. ESQ showed a significantly higher contact angle than FSU material in a water medium at all the time intervals tested and a lower contact angle than FSU in an acidic medium after 40 seconds and 60 seconds (Table 3).

**Table 3:** Pairwise comparison of contact angle among four groups BE2: Beautifil II; APX: Clearfil AP-X; FSU: Filtek Supreme Ultra; ESQ: Estelite Sigma Quick.

Pair	Water			PH5		
	20 s	40 s	60 s	20 s	40 s	60 s
BE2 vs. APX	<0.001	<0.001	<0.001	<0.001	<0.001	<0.001
BE2 vs. FSU	<0.001	<0.001	<0.001	0.140	0.243	0.923
BE2 vs. ESQ	0.984	0.470	0.635	0.110	0.165	<0.001
APX vs. FSU	<0.001	<0.001	<0.001	0.004	<0.001	<0.001
APX vs. ESQ	<0.001	<0.001	<0.001	0.005	0.048	0.001
FSU vs. ESQ	<0.001	<0.001	<0.001	0.999	0.002	<0.001

A comparison of surface roughness among the four groups showed that freshly prepared specimens showed low Sa values. APX showed the lowest value and smoothest surface while the FSU group showed the highest Sa value and the overall difference in surface roughness in the four groups was significant (Table 4).

**Table 4:** Mean and standard deviation values of surface roughness (Sa) before and after biofilm formation of the four different dental composite resins

Surface roughness (Sa)	Beautifil II (BE2)	Clearfil AP-X (APX)	Filtek Supreme Ultra (FSU)	Estelite Sigma Quick (ESQ)	p-value
Sa - before	0.37 ± 0.01	0.13 ± 0.02	0.64 ± 0.01	0.27 ± 0.01	<0.001
Sa - after	0.99 ± 0.02	0.43 ± 0.02	1.30 ± 0.14	0.74 ± 0.02	<0.001

APX group showed a significantly lower Sa value and smoother surface as compared to the other three materials while FSU showed a significantly higher Sa value and smoother surface as compared to the other three materials. BE2 material showed a significantly higher Sa value as compared to ESQ material (Table 5).

**Table 5:** Pairwise comparison of surface roughness among four groups BE2: Beautifil II; APX: Clearfil AP-X; FSU: Filtek Supreme Ultra; ESQ: Estelite Sigma Quick.

Pair	Before	After
BE2 vs. APX	<0.001	<0.001
BE2 vs. FSU	<0.001	<0.001
BE2 vs. ESQ	<0.001	<0.001
APX vs. FSU	<0.001	<0.001
APX vs. ESQ	<0.001	<0.001
FSU vs. ESQ	<0.001	<0.001

Freshly prepared specimens showed low Sa values. APX significantly revealed the lowest value and smoothest surface while BE2 showed a rougher surface but less than the ESQ group. Finally, the FSU group showed the roughest surface among the tested specimens.

Fluorescent microscopy (FM)

**Figure 2:**
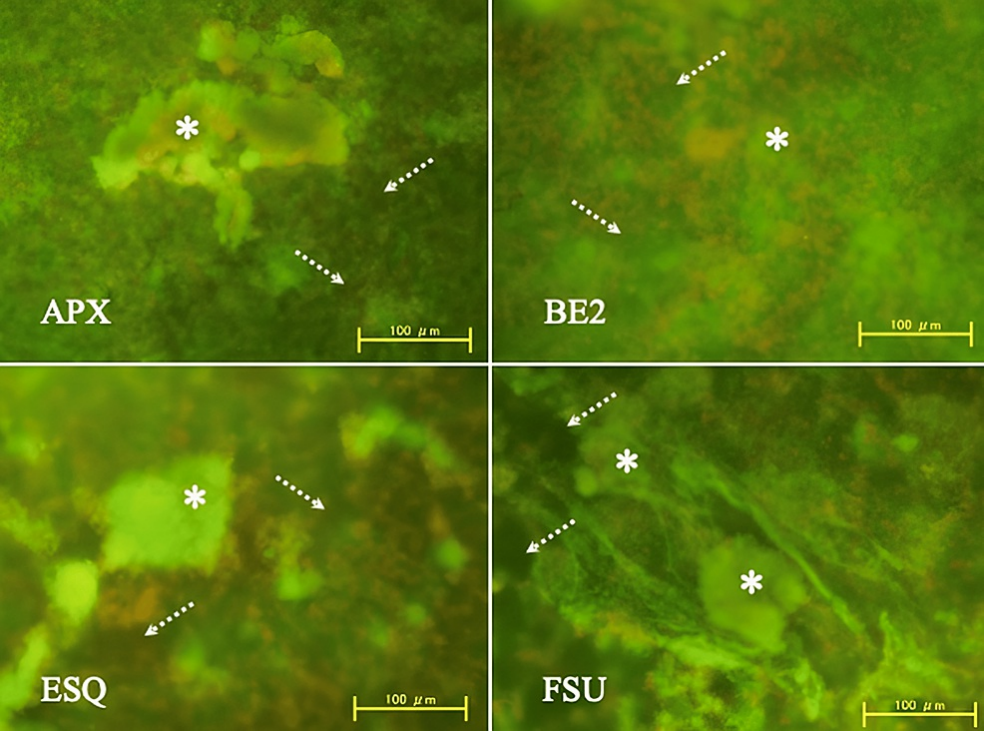
Representative fluorescence microscopic images of the S. mutans biofilms on each resin composite (original magnification x100) *: *S. mutans* colony; Arrow: material remnants; BE2: Beautifil II; APX: Clearfil AP-X; FSU: Filtek Supreme Ultra; ESQ: Estelite Sigma Quick.

After inspecting the entire biofilm on each specimen, it was confirmed that the specimens of all composites were almost completely covered by biofilms. Figure 2 describes the representative fluorescence microscopic images of the *S. mutans* biofilms on each resin composite (original magnification x100).

All specimens were almost entirely covered by biofilms that appeared mainly in green or bright-green colors on blue light excitation; small patches of dead cells appeared in orange and voids (dotted arrows) are also visible. Clusters of densely cemented biofilms (asterisks) appeared more on FSU compared to APX and ESQ; BE2 had the least on its surface. Small patches containing dead bacterial cells and voids were randomly detected. Mushroom-like clusters of densely cemented biofilms were more on FSU compared to APX and ESQ. Yet, BE2 had the least on its surface. In reverse, loosely condensed biofilms were less on FSU compared to the other three composites.

Quantitative real-time PCR observations

**Figure 3:**
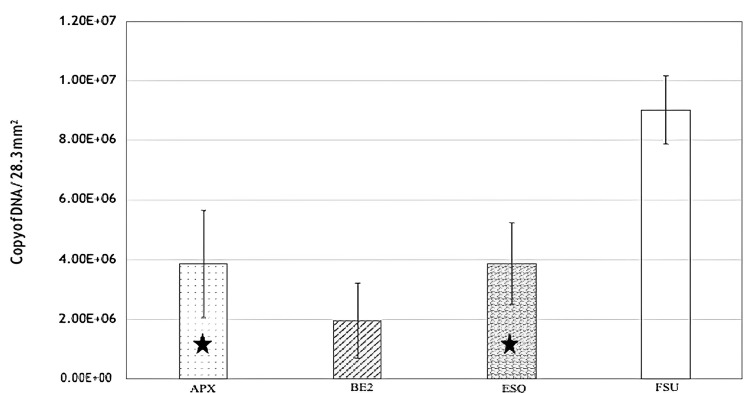
Mean and standard deviation values of DNA copies of S. mutans per specimen size (28.3 mm2) on real-time qPCR analysis BE2: Beautifil II; APX: Clearfil AP-X; FSU: Filtek Supreme Ultra; ESQ: Estelite Sigma Quick; qPCR: quantitative polymerase chain reaction.

Quantitative polymerase chain reaction analysis of biofilms produced on resin composites after only 12 hours revealed the presence of a sizable number of *S. mutans*. The average and standard deviations of *S. mutans* DNA copies per specimen size (28.3 mm2) as determined by real-time qPCR are shown in Figure 3 and Table 6.

**Table 6:** Comparison of mean and standard deviation values of DNA copies of S. mutans per specimen size (28.3 mm2) on real-time qPCR analysis BE2: Beautifil II; APX: Clearfil AP-X; FSU: Filtek Supreme Ultra; ESQ: Estelite Sigma Quick; qPCR: quantitative polymerase chain reaction.

Group	Mean	p-value	Pairwise comparison
APX	3.94 ± 1.89	<0.001	APX > BE2 (p = 0.045); APX = ESQ (p = 1.000); APX < FSU (p < 0.001); BE2 < ESQ (p = 0.048); BE2 < FSU (p < 0.001); ESQ < FSU: (p < 0.001)
BE2	1.85 ± 1.18		
ESQ	3.92 ± 1.71		
FSU	9.02 ± 1.11		

BE2 had significantly less *S. mutans* indicating the least amount of biofilm formation on its surface. APX and ESQ had almost equal quantities showing significantly higher than BE2 but significantly less than FSU, confirming that the largest amount of biofilm was formed on FSU.

**Figure 4:**
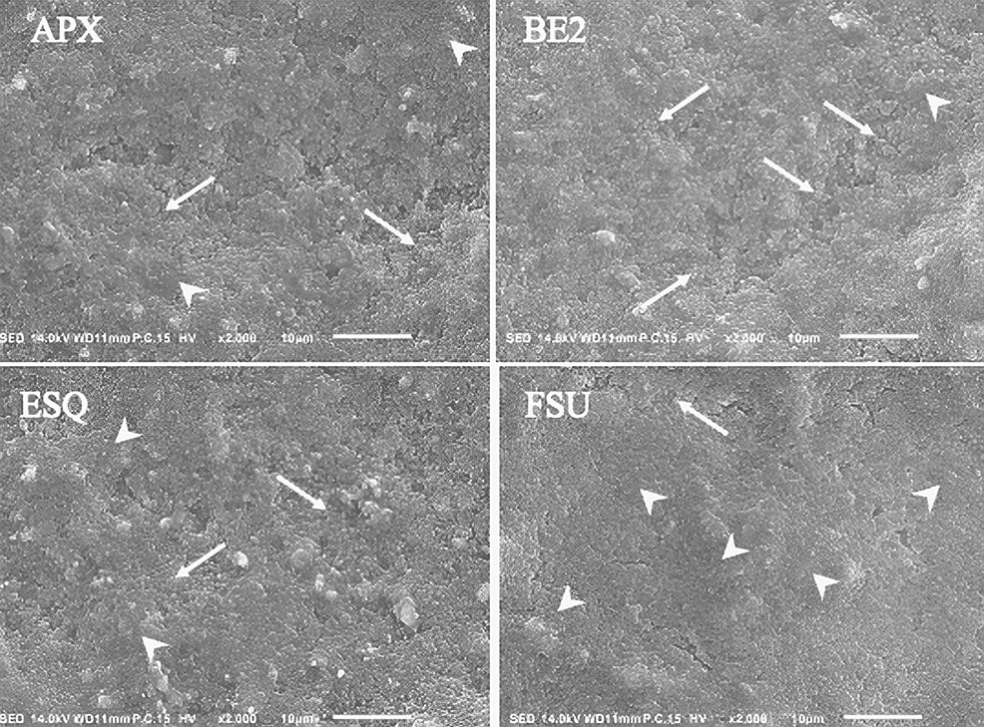
SEM photomicrographs of the biofilms at an original magnification of x2000 Arrow: biofilm imaging; BE2: Beautifil II; APX: Clearfil AP-X; FSU: Filtek Supreme Ultra; ESQ: Estelite Sigma Quick; SEM: scanning electron microscopy.

In general, all specimens were covered with biofilms having dissimilarity in their morphological patterns. All had mushroom-like highly condensed biofilm clusters growing upwards and in reverse had poorly cemented areas where *S. mutans* looked barely covered by WIG (WIG free). However, there were remarkable differences in their distribution. Figure 4 shows the SEM photomicrographs of the biofilms at an original magnification of x2000.

**Figure 5:**
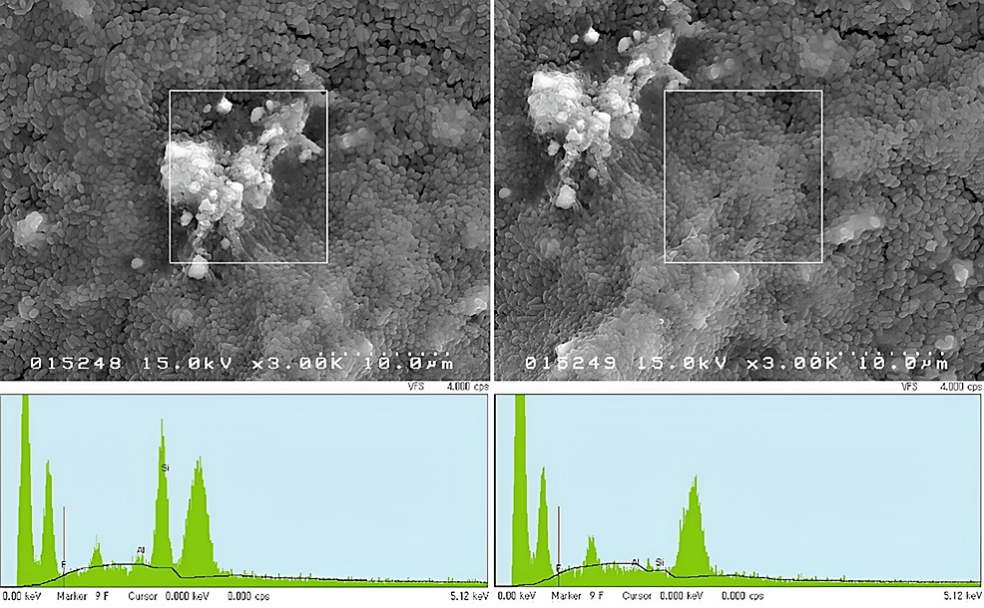
Representative EDS data from the surface of S. mutans biofilm on BE2. Upper SEM photomicrographs were taken at an original magnification of x3000 EDS: energy dispersive X-ray spectrometer; BE2: Beautifil II; SEM: scanning electron microscopy.

The representative image from each resin composite demonstrates the morphological patterns of the 12-hour *S. mutans* biofilm. Arrowheads indicate condensed parts of the biofilms cemented by thick extracellular polysaccharides (water-insoluble glucan): BE2 had the least, APX and ESQ had more compared to that, and FSU had the most. Arrows indicate areas of biofilms that *S. mutans* appeared to have minimum WIG covering them: BE2 had the most, APX and ESQ had less compared to that, and FSU had the least. BE2 had minimum condensed clusters on its surface than that on APX and ESQ. Yet, FSU had the maximum, as shown in Figure 4. Areas with WIG-free *S. mutans* were more on BE2 compared to APX and ESQ, whereas FSU had the least. There were some areas where clusters of biofilms seemingly looming upward were cemented by thick WIG on all resin composites as biofilms were continuously growing. The representative image with the original magnification of x1000 reveals each resin composite demonstrates the morphological patterns of the 12-hour *S. mutans* biofilm. Asterisks indicate condensed parts of the biofilms looming upward were cemented by thick WIG. Dotted arrows indicate exposed areas of resin composite surfaces; APX and ESQ had more of those compared to BE2 and FSU, and some glass fillers could easily be detected. Small white particles were found attached randomly on the biofilm of the BE2 surface as seen in the oval. However, some exposed areas of resin composite surfaces could also be observed. APX and ESQ had more of those exposed areas compared to BE2 and FSU. In the case of APX, some glass fillers could be observed. Interestingly, small white particles could be detected remarkably more in the biofilms of BE2 giving the impression that some chemical components had been penetrating through the biofilm. EDS peaks have indicated the presence of Si, F, and Al in those particles. Figure 5 represents the EDS data from the surface of *S. mutans* biofilm on the BE2.

Upper SEM photomicrographs were taken at an original magnification of x3000. White rectangles are the areas of EDS inspection; the left one completely covered a cluster of white particles and the right one covered a same-sized area on the biofilm near the cluster. At the bottom, EDS spectra of respective areas are shown; differences in peak sizes of Si, Al, and F are clear. Si had the largest peak, and F and Al had very small-sized peaks. EDS analysis on an area next to the cluster of white particles displayed no peak for Al and very tiny peaks for Si and F, indicating a trace amount of elements may have got mixed with the biofilm EPS. No other resin composites had such white particles accumulated at a particular area on their biofilms.

## Discussion

In this study, *S. mutans* biofilm was characterized by concentrating on one stage of biofilm formation onto four different resin composite materials. Eight to 12 hours is a critical time period for those persons who may be at high caries risks and also elderly people who may find it difficult to remove early plaque from every part of their teeth by themselves before going to sleep. In those conditions, the cumulative effect of each small episode of demineralization may be reached in an unexpectedly short time and secondary caries or root caries inevitably ensue. Simulating those practical situations, only one in vitro experimental condition was standardized using OBR in this study and compared the cured resin surfaces of four resin composites. The effectiveness and durability of cosmetic repair are significantly impacted by the presence of biofilms and the number of bacteria present. The key that the pathogens use to cause secondary caries, notably by *S. mutans*, is the buildup of plaque and subsequent colonization of bacteria on resin-composite surfaces. The formation of bacterial biofilms on restorative materials has been linked to damage and coarsening of the material's surface, leading to an increase in the number of microorganisms in the space between the restorative material and the tooth substrate, secondary dental caries, and ultimately affecting the pathology of the pulp [23,24]. Many studies have shown the correlation between bacterial adherence and the hydrophilicity of a surface. Increases in the hydrophilicity of polymeric biomaterials have been linked to dramatic rises in bacterial adherence, according to certain studies [25,26]. Despite claims from some writers that hydrophilicity reduces bacterial adherence [27,28], others have shown no association between hydrophilicity and bacterial adhesion [29-31]. FSU showed the lowest contact angle values and the most amount of biofilm in this investigation, suggesting a strong relationship between the hydrophilicity of the material and biofilm production. In this investigation, biofilm was grown on an OBR that supplied *S. mutans* with sugar. Consequently, *S. mutans* produced WIG resulting in the formation of biofilms and lowering the pH by producing organic acids, including lactic acid [32,33], which also influences the sorption process [34]. The following methods were used to reduce the bias: a uniform solidification standard and an equipment inspection technique were utilized to conduct a clinical examination of the material model disks as part of the quality control and bias prevention procedures; each material model block's solidification process and preparation procedures were tightly standardized; and to minimize system error, the same individual produced all of the sealing material disks.

In the view of conventional methacrylate-based composites, dimethacrylate polymers have been shown to exhibit a degree of conversion (DC) ranging from 55% to 75% and showing a number of monomers remained unreacted [35-37]. These monomers enhance biofilm formation [38-40]. From the previous study, FSU showed the lowest DC % (38.9 ± 6.4) among all four composites, which results in plentiful unpolymerized monomers still being present in the restoration [41]. The growth of biofilm on the resin composite surface is greatly aided by the presence of unreacted monomers in the cured composite. When oral biofilm accumulated on cured composites, the released monomers from resin composites were assumed to be retained by the biofilm matrix. Water-insoluble glucan in formed plaque may prevent these monomers from diffusing into the oral cavity. Consequently, the monomers would be trapped and condensed raising the concentration of monomers within the plaque matrix. This concentration of monomers may be high enough to enhance glucan production, even if the quantity of eluted monomers is slight at first [40]. In the same context, it was reported that dimethacrylate-based monomers such as urethane dimethacrylate (UDMA), triethylene glycol dimethacrylate (TEGDMA), and bisphenol A-glycidyl methacrylate (Bis-GMA) stimulated the growth of *S. mutans* [42]. This fact showed that uncured monomers also contribute to increased plaque accumulation on resin composite surfaces because the stimulation of bacterial growth results in more glucan production [40]. Fluoride has been incorporated in quite a good number of dental materials in recent years and studies have demonstrated its effectiveness, if not all though. BE2 contains fluorosilicate glass that has undergone an acid-base reaction before being incorporated into the resin. This matrix possesses a high amount of released fluoride ions from the pre-reacted glass (PRG), and water infiltrates into it, which leads to a greater release of fluoride [43]. The amount of fluoride released from giomers is more than that from composite resins, according to previous investigations [44,45]. Fluoride, sodium, strontium, aluminum, silicate, and boron are only some of the six ions released by the surface PRG filler in BE2. It has also been shown that the fluoride emitted from giomer affects the WIG in *S. mutans* biofilm [45], adding to the list of fluoride's antibacterial actions, which have previously been mentioned [44]. One research [46] looked at how glass-fluoride ionomer's release inhibits bacterial development and how it has a detrimental impact on adhesion. As detected in FM and SEM observations, a remarkably large number of bacterial cells remained uncovered by EPS due to less production of WIG in the case of BE2 in this study. This phenomenon may have caused due to impeded condensation of the biofilms; as a result, there was less colonization (adhesion and/or proliferation) of bacteria and that was evident in qPCR analysis too. Moreover, EDS data evidently have shown that elements have penetrated through the biofilm, have got mixed with the biofilm structure, and some ions may have penetrated into the *S. mutans* cell body causing downregulation of metabolic activities resulting in less production of WIG.

In comparison to BE2, APX and ESQ had significantly more amount of bacteria according to qPCR data; however, on FM and SEM observations, notable areas of biofilm-free surfaces could be detected. This phenomenon might be an effect of the initial surface roughness (Sa) of the materials that might have hindered the bacterial adhesion at the very starting stage or might have detached partially when samples were washed with PBS before analysis. Sa of all cured resin surfaces of the resin composites increased significantly (by about three times) from that of the initial stage as pH dropped from 7.3 to 4 in about six hours and were at that condition for more than five hours as monitored in real time (data not shown). Organic acids and bacterial enzymes are believed to be the reasons causing the damage. Interestingly, Sa value after biofilm formation showed significant differences between the resin composites; APX showed the least deterioration, while FSU had the most. BE2 was next to FSU and that might be due to the release of ions including F. The findings of this study have clearly demonstrated the differences in biofilms formed on the four dental resin composite surfaces for 12 hours. Most importantly, significant differences in the copy of DNA using a real-time PCR technique were found among all the experimental groups except between APX and ESQ. This was supported by the microscopic and morphological findings. Thus, the null hypothesis tested in this study was rejected.

In addition, this study can be an in vitro model for quantitative and qualitative analysis with an optimum amount of biofilms that are required to acquire non-biased data of amplified DNA copies by a real-time PCR machine and thereafter display differences significantly between the experimental groups with reproducibility. The overgrowth of biofilms produced over a longer period of time makes it difficult to find any differences between the substrates where the quality of the material or surface condition generally loses its influence.

The present study, like other research, has a main limitation. The differences in the resin monomers at the composite materials were not evaluated because of the lack of TEGDMA and Bis-GMA actual percentage, which was held by manufacturers as trade secrets. Yet, this study offers insight into the behavior and the assessment of *S. mutans *early biofilm formation on four different cured dental composite resin surfaces.

## Conclusions

From a dental public health stance, composite resins have been used over the past decades to restore individuals’ dental functions and esthetics and improve patients’ oral health and quality of life, leading dental practitioners to plan successful dental care. The findings of this study showed that the differences in early biofilm formation on the resin surfaces of the four resin composites are dependent on the differences in material compositions and their surface properties. This study suggested that biofilm would form differently on resin composite and giomer. BE2 showed the lowest quantity of biofilm accumulation compared to other resin composites (APX, ESQ, and FSU). This finding could be attributed to the fact that the BE2 is a giomer and contains fluoride, which may be the main reason for the lowest quantity of biofilm accumulation. Continued studies and future research that examine this territory of research in relation to *S. mutans* early biofilm formation on different restorative materials are required. Exploring this area of research might not only be helpful and supportive for researchers or clinicians, but it might also be important for the production of improved quality of dental composite materials, which will certainly impact the quality of the delivered dental care and the patients’ oral health and quality of life.
